# Inhibition of Hydrogen Sulfide Production by Gene Silencing Attenuates Inflammatory Activity by Downregulation of NF-*κ*B and MAP Kinase Activity in LPS-Activated RAW 264.7 Cells

**DOI:** 10.1155/2014/848570

**Published:** 2014-08-19

**Authors:** Alireza Badiei, Nethaji Muniraj, Stephen Chambers, Madhav Bhatia

**Affiliations:** Department of Pathology, University of Otago-Christchurch, P.O. Box 4345, Christchurch 8140, New Zealand

## Abstract

Hydrogen sulfide is an endogenous inflammatory mediator produced by the activity of cystathionine *γ*-lyase (CSE) in macrophages. The objective of this study was to explore the mechanism by which hydrogen sulfide acts as an inflammatory mediator in lipopolysaccharide- (LPS-) induced macrophages. In this study, we used small interfering RNA (siRNA) to inhibit CSE expression in macrophages. We found that CSE silencing siRNA could reduce the LPS-induced activation of transcription factor nuclear factor-*κ*B (NF-*κ*B) significantly. Phosphorylation and activation of extra cellular signal-regulated kinase 1/2 (ERK1/2) increased in LPS-induced macrophages. We showed that phosphorylation of ERK in LPS-induced RAW 264.7 cells reached a peak 30 min after activation. Our findings show that silencing CSE gene by siRNA reduces phosphorylation and activation of ERK1/2 in LPS-induced RAW 264.7 cells. These findings suggest that siRNA reduces the inflammatory effects of hydrogen sulfide through the ERK-NF-*κ*B signalling pathway and hydrogen sulfide plays its inflammatory role through ERK-NF-*κ*B pathway in these cells.

## 1. Introduction

Hydrogen sulfide is a toxic gas with a foul odour. It is also now generally accepted that it acts as a gaseous signalling transmitter along with carbon monoxide (CO) and nitric oxide (NO). Hydrogen sulfide is produced* in vivo* from L-cysteine predominately by cystathionine gamma-lyase (CSE), cystathionine beta synthase (CBS), and 3-mercaptopyruvate sulfurtransferase (3-MST) [1]. Expression of CSE in the periphery [2] and CBS in the central nervous system [3, 4] determines which enzyme is involved in hydrogen sulfide production at these sites. It has been reported that hydrogen sulfide is produced by activity of CSE in macrophages and following this production hydrogen sulfide would induce further activation of macrophages [5–8] suggesting an important role for hydrogen sulfide in the signalling of inflammation.

Inflammation is a defensive mechanism induced by the host in response to injury or infection. Macrophages play a crucial role during inflammation. Activated macrophages have been shown to amplify inflammation via release of proinflammatory mediators such as tumour necrosis factor-*α* (TNF-*α*), interleukin-6 (IL-6), and interleukin-1*β* (IL1-*β*). Cytokine secretion induced by hydrogen sulfide has been reported in an* in vitro* study of human monocytes [9] suggesting an important role for hydrogen sulfide in inflammatory signalling.

Hydrogen sulfide acts as an inflammatory mediator and its levels increase during inflammation. Both exogenously administrated and high level endogenously generated hydrogen sulfides exert proinflammatory activity. It has been reported that hydrogen sulfide may play a proinflammatory role in acute pancreatitis [10, 11], burn injury [12], lipopolysaccharide- (LPS-) induced endotoxemia [13], hemorrhagic shock [14], human monocytes [9], and mouse macrophage [5]. Inhibition of hydrogen sulfide production with a CSE inhibitor, DL-propargylgycine (PAG) [15], and with a CSE gene silencer, small interfering RNA (siRNA) [5], has been shown to ameliorate inflammation suggesting of endogenous hydrogen sulfide has an important role in the pathophysiology of inflammation.

Macrophages produce and release a wide range of proinflammatory mediators in response to LPS treatment. This depends on inducible gene expression mediated by the activation of mitogen-activated protein kinase (MAPK) and the transcription factor nuclear factor-*κ*B (NF-*κ*B) [16–18].

NF-*κ*B and MAPK cascades play key roles in intracellular signalling pathways involved in cytokines production. Three families of MAPK including c-Jun N-terminal kinase (JNK), extracellular-signal-regulated kinase (ERK), and p38 regulate many genes particularly those of the inflammatory and immune response [19].

Inducible NF-*κ*B regulates transcription of immune and inflammatory response genes [20]. NF-*κ*B is implicated in activation of gene expression for various proinflammatory mediators including TNF-*α*, IL-6, IL-1*β*, and monocyte chemoattractant protein-1 (MCP-1) [21–24].

ERK plays an important role in the temporal control of NF-*κ*B transcriptional activity and expression of NF-*κ*B-regulated genes, as well as an upstream activator of NF-*κ*B [25, 26]. Inhibition of ERK reduced NF-*κ*B transcription activity, suppressed the transcription of NF-*κ*B-dependent genes, and protected against LPS-induced endotoxemia [25, 27, 28]. Hydrogen sulfide was shown to activate macrophages with expression and production of proinflammatory cytokines. Strong activation of ERK but not p38 MAPK and JNK was reported in macrophages treated with hydrogen sulfide [29]. Inhibition of ERK resulted in hydrogen sulfide-dependent increase in NF-*κ*B activity. Therefore, ERK plays an important role in signal transduction of macrophages treated with hydrogen sulfide [29]. Hydrogen sulfide was also shown to regulate the inflammatory response in a mice model of sepsis via activation of the ERK [30]. An* in vitro* study showed that overexpression of CSE sustainably activates ERK1/2 and p38 MAPK phosphorylation [31]. Zhang and coworkers showed that hydrogen sulfide regulates inflammatory response in a cecal ligation puncture (CLP) model of sepsis through activation of the ERK pathway [30].

In this study, we assess the mechanism by which CSE targeting siRNA downregulates the inflammatory status of monocytes and examine the role of NF-*κ*B and MAPKs in this process.

## 2. Materials and Methods

### 2.1. Cell Line and Treatments

The murine macrophage cell line, RAW 264.7 cells, was maintained at 37°C in Dullbecco's modified Eagle's medium (DMEM; Gibco BRL, USA) containing 10% heat inactivated fetal bovine serum (FBS; Gibco BRL, USA) supplemented with penicillin (100 U/mL; Gibco BRL, USA) and streptomycin (100 *μ*g/mL; Gibco BRL, USA). Preliminary experiments demonstrated (data not shown) that the levels of cytokine production were at their optimum 24 h after administration of LPS (Sigma, Cat. number L-2630) at a concentration of 100 ng/mL. This treatment strategy was therefore used for subsequent experiments in this study.

### 2.2. siRNA Transfection

Reverse transfection was performed with Lipofectamine RNAiMAX (Invitrogen) as described previously [5]. Briefly, cells (7 × 10^5^/well) were seeded into a 6-well plate. At 24 h after transfection media were replaced with standard media containing serum and antibiotic, and wells designated to the activated group were treated with LPS. Transfection efficiency was confirmed using Block-iT Alexa Fluor Red Fluorescent Oligo (Invitrogen) as per the manufacturer's instructions. We used 50 nM siRNA in our experiments to silence CSE gene expression.

### 2.3. Nuclear Protein Extraction from Cells

Nuclear protein was extracted according to the protocol from Sigma for nuclear protein extraction without the use of detergent, with minor modifications. The following buffers were prepared to extract the nuclear protein: hypotonic lysis buffer: 10 mM HEPES (pH 7.9), 1.5 mM MgCl_2_, 10 mM KCl, 0.1 DTT, and protease inhibitor cocktail; extraction buffer: 20 mM HEPES (pH 7.9), 1.5 mM MgCl_2_, 0.42 mM NaCl, 0.2 mM EDTA, 25% glycerol, 0.1 DTT, and protease inhibitor cocktail. Briefly, cells were washed twice with PBS, scraped using fresh PBS, and then centrifuged for 5 min at 450 g. Pellets were resuspended using 400 *μ*L of lysis buffer, incubated for 15 min at 4°C, and centrifuged for 5 min at 420 g. The pellets were resuspended in 250 *μ*L lysis buffer and passed through a number-20 gauge needle using a single rapid stroke. Cells were then centrifuged for 20 min at 5000 g. The pellets were resuspended with 60 *μ*L of extraction buffer, shaken for 30 min at 600 g at RT, and centrifuged for 5 min at 11000 g. The supernatants were transferred into fresh tubes and stored at −70°C. A DC protein assay (Bio-Rad) was used to determine the protein concentration.

### 2.4. NF-*κ*B Activity Assay

The binding of NF-*κ*B to DNA was measured in nuclear extract using an ELISA based TransAmTM NF-*κ*B p65 transcription factor assay kit (Active Motif) as per the manufacturer's instructions. Briefly, nuclear extracts (20 *μ*g) were incubated in a 96-well plate with complete lysis buffer for 1 h followed by incubation with a specific primary antibody against NF-*κ*B p65 for 1 h. Subsequently, a horse radish peroxidase- (HRP-) conjugated secondary antibody was used for detection. The enzymatic product was measured at 450 nm using a microplate reader. The specificity of the assay was determined by wild-type or mutated comparative control wells. Results were expressed as fold increase over control groups.

### 2.5. Western Blot

Nuclear extract proteins were separated on 10% SDS-polyacrylamide gels and subsequently transferred to PVF membranes. The membranes were blocked for 1 h in 5% BSA in Tris-buffered saline containing 0.01% Tween 20 (TBST). Immunoblotting was performed overnight at 4°C with primary antibodies, anti-ERK1/2, and anti-phospho-ERK1/2 (Cell Signalling) at a dilution of 1 : 2000 and 1 : 1000, respectively. Membranes were incubated with a secondary horseradish peroxidase-conjugated antibody at 1 : 10000 dilutions for 1 h at room temperature. To visualize immunoreactive proteins, the enhanced chemiluminescence detection kit (PerkinElmer, USA) was used. Hypoxanthine-guanine phosphoribosyl transferase (HPRT) (Santa Cruz Biotechnology) was used as the housekeeping protein.

### 2.6. Statistics

To analyse the data one way ANOVA and Tukey's multiple comparison tests were used. The experiments were performed in triplicate and the results are expressed as mean ± SD. *P* values <0.05 were regarded as statistically significant.

## 3. Results

### 3.1. Inhibition of NF-*κ*B Activity by siRNA in RAW 264.7 Cells

**Figure 1:**
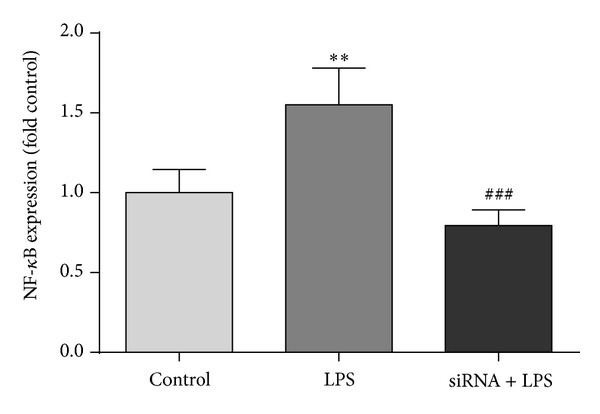
The effect of LPS and siRNA on NF-*κ*B activity. NF-*κ*B activity is expressed as a fold control. The control group is untreated, the LPS group has received 100 ng/mL LPS 24 h prior to assay, and the LPS + siRNA group has received 50 *μ*M siRNA 24 h prior to LPS treatment which was administrated 24 h prior to assay. For all groups *N* = 3. Error is SD. ∗∗*P* < 0.01 compared to control and ^###^ *P* < 0.001 compared to LPS using planned comparisons.

The NF-*κ*B pathway is essential for the activation of most inflammatory genes. To examine whether the NF-*κ*B signal transduction pathway is involved in LPS-induced CSE expressions, RAW 264.7 cells were stimulated with LPS following treatment with siRNA. Analysis of the NF-*κ*B DNA binding assay revealed that the levels of the NF-*κ*B activity in the LPS-induced (100 ng/mL) RAW 264.7 cells were significantly higher than untreated cells. NF-*κ*B DNA binding assay analysis demonstrated that the levels of NF-*κ*B activity in the LPS-induced (100 ng/mL) RAW 264.7 cells pretreated with siRNA reduced significantly compared to untreated cells. There was a significant effect of LPS and siRNA treatment on NF-*κ*B expression in RAW 264.7 macrophages (*F* (2, 9) = 21.85; *P* < 0.001) (Figure 1). LPS treatment 24 h prior to NF-*κ*B DNA binding assay resulted in a significantly higher level of NF-*κ*B expression with levels 1.5-fold (0.23 SD) higher than control (*P* < 0.01) (Figure 1). siRNA administration reduced the effect of LPS on NF-*κ*B expression to 0.8-fold (0.9 SD) lower than control (*P* < 0.001) (Figure 1).

### 3.2. Effect of CSE Targeting siRNA on the Phosphorylation of MAP Kinase

Activated macrophages are induced by three families of MAP kinase. These kinase families play an important role in cell growth, stress induced gene expression, and differentiation [32, 33].

**Figure 2:**
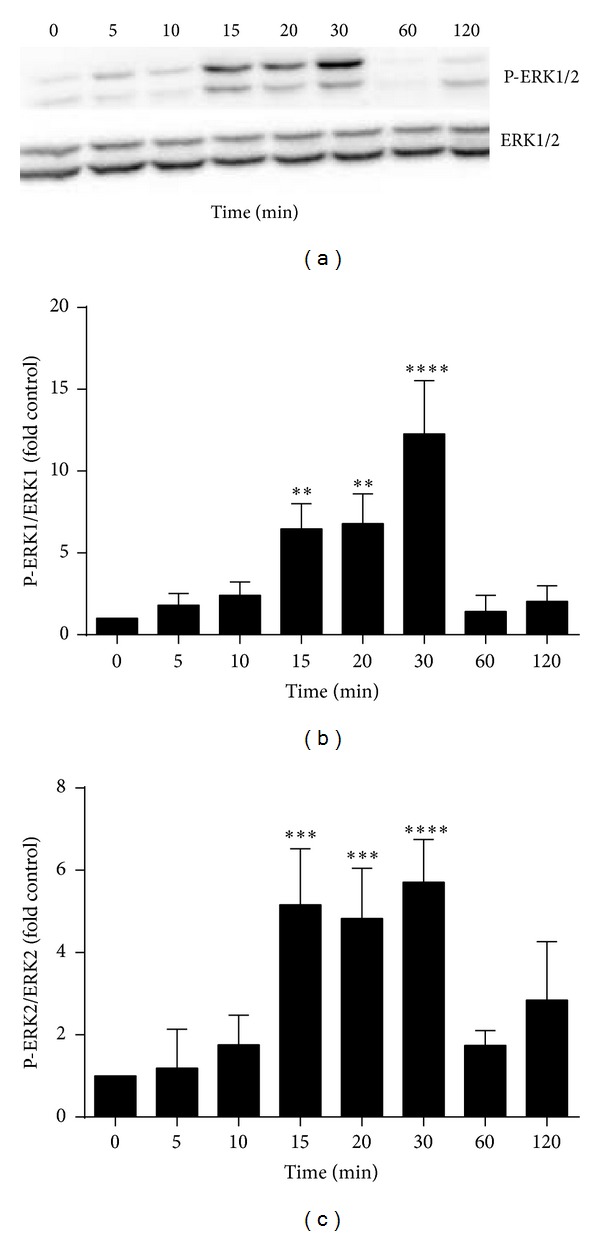
LPS induces phosphorylation of ERK1/2 in a time-dependent manner. RAW 264.7 cells were incubated with 100 ng/mL LPS for 0–24 h. Total cell lysates were examined by western blot with anti-ERK1/2 and phosphor-ERK1/2 (P-ERK) antibodies. (a) Results shown are representative blots from three different experiments. (b and c) The histograms represent the optical density of phosphor-ERK1/2: total ERK1/2 expressed as control fold increase. Results shown are the mean ± SD of the three experiments. ∗∗∗∗*P* < 0.0001, ∗∗∗*P* < 0.001, and ∗∗*P* < 0.01 compared to control.

Before the siRNA experiments could be performed in the time course of phosphorylation following LPS activation, the phosphorylation needed to be established. To elucidate this, an experiment was performed where LPS was administered at various time periods and cell lysates were analysed for phosphorylation of ERK1/2. Our results showed that the enhanced phosphorylation of ERK1/2 was found as early as 15 min after treating cells with LPS and persisted for 15 min (Figure 2).

**Figure 3:**
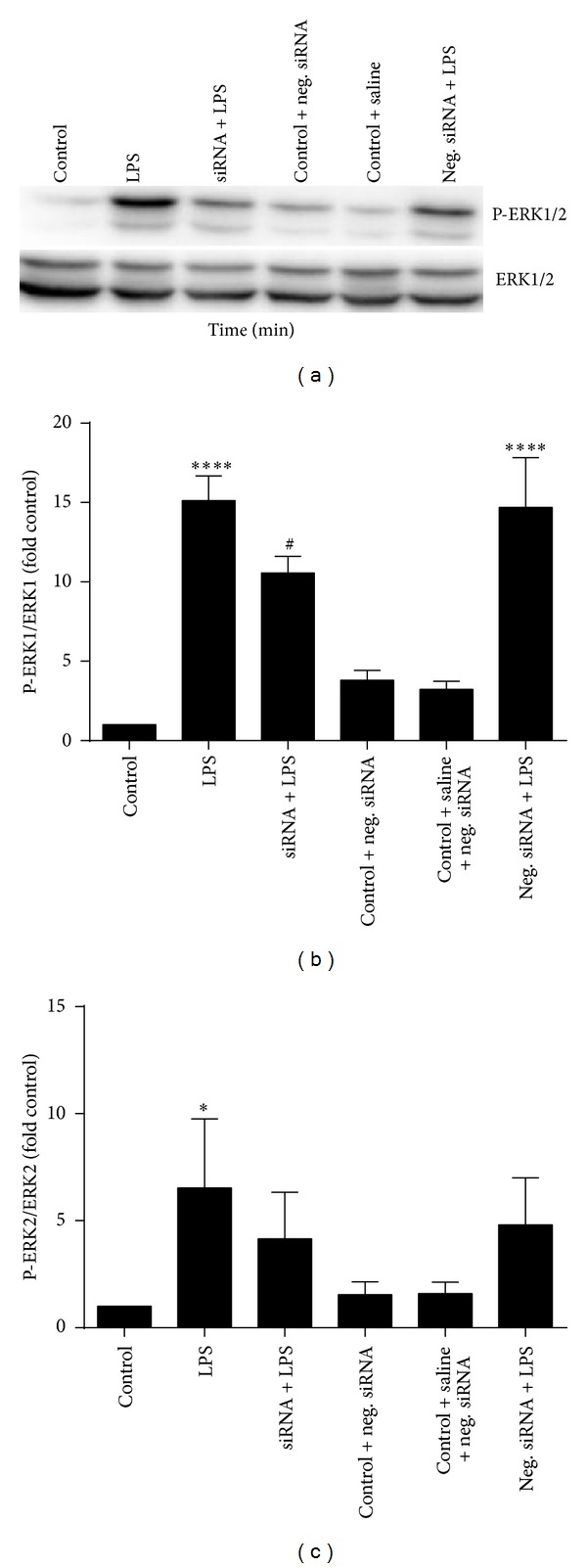
The effect of siRNA on the phosphorylation of ERK1/2 in RAW 264.7 cells macrophages. Cells were incubated with 100 ng/*μ*L LPS for 30 min and cell lysates were examined by western blot using anti-ERK1/2 and phosphor-ERK1/2 (P-ERK) antibodies. (a) Results shown are representative blots from three independent experiments. (b and c) The histograms represent the ratios of the optical density of phosphor-ERK1/2: total ERK1/2 expressed as fold control. Data shown are the mean ± SD of the three experiments.

Using this time point (30 min), we examined the effect of CSE targeting siRNA on the phosphorylation of ERK MAP kinase. Our results showed that siRNA targeting CSE inhibited LPS-induced phosphorylation of ERK1/2 (Figure 3). These results revealed that siRNA has inhibitory effects on the production of the inflammatory mediators through inhibition of MAP kinase ERK1/2 phosphorylation.

## 4. Discussion

Hydrogen sulfide is an inflammatory mediator with an important role in normal physiology and disease. Elevated levels of hydrogen sulfide [34] and increased CSE mRNA expression [5, 8] following LPS administration have been shown. In a previous study [5] we demonstrated that transfection of siRNA in RAW 264.7 cells resulted in lower levels of CSE mRNA and protein expression as well as reduced levels of proinflammatory mediators. In this study, the mechanism by which hydrogen sulfide acts as a proinflammatory mediator was examined using LPS-activated RAW 264.7 cells pretreated with siRNA. In this experiment, we used 50 nM siRNA to silence CSE gene expression. Cell viability assay showed that this dose of siRNA had no cytotoxic effects on RAW 264.7 cells [5].

Treatment of macrophages with LPS triggers the activation of various signalling pathways among which the MAPKs family play a significant role in inflammation. Phosphorylation of ERK increased as early as 15 min after LPS treatment and reached a peak at 30 min which was in accordance with previous reports [35, 36]. However, it is of interest to note that inhibition of hydrogen sulfide production by silencing the CSE gene using siRNA in cells treated with LPS for 30 min resulted in reduced phosphorylation of ERK.

NF-*κ*B plays an important role as a central transcription factor regulating the transcription of many proinflammatory cytokines, chemokines, and adhesion molecules. NF-*κ*B is present in the cytoplasm where it exists in an inactive state, bound to an inhibitory protein *κ*B (I*κ*B). Upon activation, NF-*κ*B dissociates from I*κ*B and translocates into the nucleus where it binds to 10-bp sequences in the regulatory regions of several inflammatory genes, upregulating their transcription [37, 38].

Recently, NF-*κ*B has been shown to be involved in TLR4-mediated upregulation of CSE mRNA expression and generation of hydrogen sulfide in LPS-induced macrophages [36]. In accordance with this report, our findings also suggest that NF-*κ*B plays a key role in the inflammatory response of LPS-induced macrophages. In this study, we also demonstrated that inhibition of CSE gene expression by siRNA resulted in a decreased NF-*κ*B activation in LPS-induced macrophages. In a previous report, we have demonstrated the downregulation of inflammatory mediators by silencing the CSE gene with siRNA. Our findings show that siRNA targeting CSE reduced inflammation status by reducing the activity of NF-*κ*B. These findings indicate that inhibition of proinflammatory mediator production by LPS-induced RAW 264.7 cells may involve transcriptional regulation through suppression of NF-*κ*B-DNA binding potential and interference with nuclear translocation of NF-*κ*B.

Previously it has been shown that hydrogen sulfide could activate NF-*κ*B in human monocyte cells, U937 cells, via degradation of I*κ*B-*α* [9]. The results of our present study are consistent with this report as we found that hydrogen sulfide and LPS-induced RAW 264.7 cells increase the activity of NF-*κ*B, and this effect was attenuated by siRNA against CSE. Therefore, hydrogen sulfide induces NF-*κ*B activation and acts as a proinflammatory mediator in macrophages.

In conclusion, this study shows that CSE-targeted siRNA inhibited NF-*κ*B activation and ERK phosphorylation in LPS-induced macrophages and, consequently, downregulates proinflammatory mediator secretion by LPS-induced macrophages. This indicates that hydrogen sulfide acts as an inflammatory mediator through ERK-NF-*κ*B pathway.
